# Paeoniflorin Ameliorates Spinal Cord Injury by Controlling Apoptosis and Ferroptosis in H_2_O_2_‐Damaged PC12 Cells

**DOI:** 10.1002/iid3.70324

**Published:** 2026-01-20

**Authors:** Zongyu Zhang, Zhijing Zhou, Peng Zhang, Yongfeng Huo

**Affiliations:** ^1^ Department of Orthopedics Lianyungang Affiliated Hospital of Nanjing University of Chinese Medicine, The Traditional Chinese Medical Hospital of Lianyungang Lianyunagng China; ^2^ Department of Orthopedics The Affiliated Lianyungang Hospital of Xuzhou Medical University/The First People's Hospital of Lianyungang Lianyungang China

**Keywords:** apoptosis, ferroptosis, oxidative stress, paeoniflorin, SIRT3, spinal cord injury

## Abstract

**Background:**

Spinal cord injury (SCI) leads to severe neurological dysfunction. Current therapeutic strategies remain limited, with poor recovery rates. Oxidative stress and ferroptosis are key mechanisms underlying secondary SCI. Paeoniflorin has anti‐inflammatory, antioxidant, and neuroprotective properties; however, its role in regulating apoptosis and ferroptosis after SCI remains unclear.

**Methods:**

An *in vitro* SCI model was established by treating PC12 cells with 300 μM H₂O₂ for 24 h, followed by intervention with various concentrations of paeoniflorin. Cell viability was assessed using the 3‐(4, 5‐dimethylthiazol‐2‐yl)‐2, 5‐diphenyl tetrazolium bromide (MTT) assay, apoptosis was analyzed by flow cytometry, lipid reactive oxygen species (ROS) levels were detected by immunofluorescence, and cysteine (Cys), glutathione (GSH), and glutathione peroxidase 4 (GPX4) levels were measured using enzyme‐linked immunosorbent assay (ELISA) kits. Western blotting and reverse transcription quantitative polymerase chain reaction (RT‐qPCR) were performed to evaluate the expression of sirtuin 3 (SIRT3), B‐cell lymphoma‐2 (Bcl‐2), and BCL2‐Associated X (Bax). In addition, the SIRT3‐specific inhibitor, 3‐TYP, was used to validate the role of SIRT3 in paeoniflorin‐mediated protection.

**Results:**

Paeoniflorin increased cell viability; reduced apoptosis; suppressed ROS accumulation; and restored Cys, GSH, and GPX4 levels in a dose‐dependent manner. Paeoniflorin significantly upregulated SIRT3 mRNA and protein expression. Co‐treatment with 3‐TYP attenuated the protective effects of paeoniflorin, indicating that the role of paeoniflorin is mediated through activation of the SIRT3 pathway.

**Conclusion:**

Paeoniflorin exerts significant neuroprotective effects against SCI‐induced injury by activating the SIRT3 signaling pathway and regulating apoptosis, oxidative stress, and ferroptosis, offering a novel potential therapeutic target for SCI treatment.

## 1 Introduction

Spinal cord injury (SCI) is a common and severe form of central nervous system injury primarily caused by traumatic factors such as traffic accidents, falls, and acts of violence, leading to motor, sensory, and autonomic dysfunction below the level of injury [1, 2, 3]. Globally, approximately 23 cases of SCI are recorded per million individuals annually, predominantly affecting young adults, and resulting in a high disability rate, decreased quality of life, and significant social and economic burdens [4, 5]. Although various interventions, including surgery, pharmacotherapy, and stem cell therapy, have been applied [6, 7], the complex pathophysiology of SCI continues to limit treatment outcomes, and effective prevention and management remain considerable challenges.

The pathological process of SCI involves both the primary and secondary injury phases. Primary injury refers to the direct mechanical disruption of the spinal cord tissue, whereas secondary injury is defined by a series of processes that occur continuously in the first stages, including the production of free radicals, delayed calcium influx, immune and inflammatory system responses, and cell death [8, 9]. Among these, oxidative stress is a key contributor to secondary injury progression. Excessive reactive oxygen species (ROS) production damages neuronal nucleic acids, proteins, and lipids, and further promotes neuroinflammation and the activation of various forms of programmed cell death, thereby exacerbating neurological dysfunction [10]. Recent studies have identified ferroptosis as an iron‐dependent, lipid peroxidation‐driven form of programmed cell death that plays a crucial role in secondary SCI pathology [11, 12]. The characteristic features of ferroptosis include mitochondrial shrinkage, increased membrane density, and ROS accumulation [13, 14]. Animal experiments have demonstrated that treatment with the ferroptosis inhibitor ferrostatin‐1 significantly improves motor function in SCI mouse models [15], suggesting that ferroptosis may serve as a novel therapeutic target for SCI. Therefore, targeting ferroptosis and exploring new cytoprotective strategies may lead to important theoretical and therapeutic advances in SCI management.

In the search for effective interventions targeting secondary injuries following SCI, natural bioactive compounds have attracted increasing attention because of their multi‐target effects and low toxicity profiles. Paeoniflorin, a monoterpene glycoside extracted from the roots of *Paeonia lactiflora* or *Paeonia veitchii*, is a major active constituent widely used in traditional Chinese medicine [16, 17, 18]. Extensive studies have demonstrated that paeoniflorin possesses multiple biological activities, including anti‐inflammatory, antioxidant, immunomodulatory, antiapoptotic, and neuroprotective effects [17, 19]. Early investigations reported that paeoniflorin improved learning and memory deficits in animal models of dementia [20]. Subsequently, paeoniflorin was found to exert neuroprotective effects by inhibiting signaling pathways such as NF‐κB and vascular endothelial growth factor (VEGF)/VEGF receptor‐1 (Flt‐1), and showed therapeutic potential in models of Alzheimer's disease, cerebral ischemia, and Parkinson's disease [21, 22, 23, 24]. Recent studies have shown that paeoniflorin significantly alleviates neuropathic pain in chronic constriction injury rat models by inhibiting spinal glial activation and MAPK signaling pathways [25] and by activating the Keap1‐Nrf2 pathway to reduce ROS levels and suppress NLRP3 inflammasome activation, thereby mitigating neuroinflammation [26, 27]. Although substantial evidence supports the neuroprotective role of paeoniflorin in various neurological disorders, its effects on motor function recovery after SCI and the regulatory mechanisms involving ferroptosis remain largely unexplored.

The aim of this study was to elucidate the role and underlying mechanisms of paeoniflorin in SCI, with the goal of providing new theoretical insights for the development of SCI therapies.

## 2 Materials and Methods

### 2.1 Cell Culture and Treatment

PC12 cells (pheochromocytoma cells) were obtained from the Cell Bank of the Chinese Academy of Sciences (Shanghai, China). Cells were cultured in Dulbecco's modified Eagle's medium (Gibco, USA) supplemented with 10% heat‐inactivated fetal bovine serum (Gibco, USA) and 1% penicillin‐streptomycin at 37°C in a humidified atmosphere containing 5% CO₂.

To establish an oxidative stress injury model, PC12 cells were pretreated with 300 μM hydrogen peroxide (H₂O₂; Sigma‐Aldrich, USA) for 24 h. After H₂O₂ exposure, cells were treated with different concentrations of paeoniflorin (25, 50, and 100 μM; MedChemExpress, USA) for an additional 24 h. In specific experiments, cells were co‐treated with paeoniflorin (100 μM) and the sirtuin 3 (SIRT3) inhibitor 3‐TYP (30 μM; Selleck Chemicals, USA).

### 2.2 Cell Viability Assay

Cell viability was assessed using the MTT assay. Briefly, PC12 cells were seeded in 96‐well plates and subjected to the indicated treatments. After treatment, 10 μL of MTT solution (5 mg/mL; C0009S, Beyotime) was added to each well and incubated at 37°C for 4 h. Subsequently, the medium was removed, and 150 μL of dimethyl sulfoxide was added to dissolve the formazan crystals. Absorbance was measured at 570 nm using a microplate reader (BioTek, USA).

### 2.3 Flow Cytometry Analysis of Apoptosis

Apoptosis was evaluated using Annexin V‐FITC/PI apoptosis detection kits (40302ES50; Yeasen Biotechnology [Shanghai] Co. Ltd.) according to the manufacturer's instructions. Treated PC12 cells were harvested, washed with PBS, and stained with Annexin V‐FITC and propidium iodide (PI) in the dark for 15 min. Samples were analyzed using a flow cytometer (BD FACSCanto II, USA) and FlowJo software (version 10.8.1, Becton, Dickinson, and Company).

### 2.4 Western Blotting

Total protein was extracted using RIPA buffer (P0013B; Beyotime, Shanghai, China) supplemented with protease inhibitors (Roche). Protein concentrations were determined using a BCA assay kit (P0010S; Beyotime, Shanghai, China). Equal amounts of protein (40 μg) were separated by SDS‐PAGE and transferred to PVDF membranes (Millipore, USA). Membranes were blocked with 5% nonfat milk and incubated overnight at 4°C with primary antibodies against SIRT3 (10099‐1‐AP; Proteintech, China), Bax (50599‐2‐Ig; Proteintech), Bcl‐2 (12789‐1‐AP; Proteintech), and GAPDH (60004‐1‐Ig; Proteintech). After incubation with HRP‐conjugated secondary antibodies, bands were visualized using an enhanced chemiluminescence (ECL, P2300, New Cell&Molecular Biotech Co. Ltd.) reagent and quantified using ImageJ software (National Institutes of Health).

### 2.5 Detection of ROS

Intracellular ROS levels were measured using a DCFH‐DA fluorescent probe (S0033; Beyotime). After treatment, the cells were incubated with 10 μM DCFH‐DA at 37°C for 30 min in the dark. Following washing with PBS, the fluorescence intensity was observed under a fluorescence microscope (Olympus, Japan) and quantified using ImageJ software.

### 2.6 Enzyme‐Linked Immunosorbent Assay (ELISA) Assay

The levels of cysteine (Cys, CN‐E‐BC‐K352‐M), glutathione (GSH, CN‐E‐BC‐K030‐M), and glutathione peroxidase 4 (GPX4, CN‐E‐BC‐K883‐M) were determined using ELISA kits (Elabscience, China) following the manufacturer's instructions. The absorbance was measured using a microplate reader.

### 2.7 Reverse Transcription Quantitative Polymerase Chain Reaction (RT‐qPCR) Analysis

Total ribonucleic acid (RNA) was extracted using TRIpure Total RNA Extraction Reagent (YFXM0011P, Yi Fei Xue Biotechnology, China) and reverse‐transcribed into cDNA using HiScript III RT SuperMix for qPCR (R323, Vazyme, China). RT‐qPCR was performed using the ChamQ Universal SYBR qPCR Master Mix (Q711, Vazyme, China) on a CG Real‐Time PCR system (Heal Force). The relative expression levels of target genes were normalized to that of β‐actin and calculated using the 2^−ΔΔCt^ method [28]. All experiments were performed in triplicate to ensure reproducibility. The primer sequences for the target genes are listed in Table 1.

**Table 1:** The RT‐qPCR primer sequences.

Name	Sequence (5′‐3)	
GAPDH	Sense	AGGTCGGAGTCAACGGATTT
	Antisense	TGACGGTGCCATGGAATTTG
SIRT3	Sense	actactttctccggctgctt
	Antisense	acaatgtcgggcttcacaac

### 2.8 Statistical Analysis

Statistical analyses were conducted using GraphPad Prism 8.0 software. Differences between groups were analyzed using one‐way ANOVA followed by Tukey's post‐hoc test. All experiments were performed in triplicate. Data are presented as mean ± standard deviation (SD). Statistical significance was set at *p* < 0.05.

## 3 Results

### 3.1 Effect of Paeoniflorin on Cell Viability in H₂O₂‐Damaged PC12 Cells

**Figure 1:**
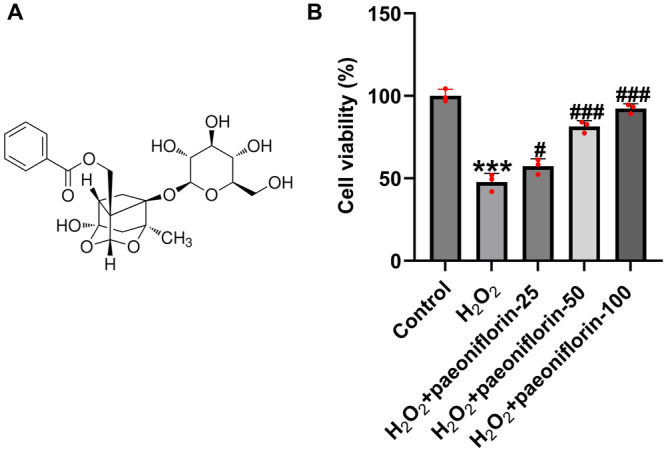
Effect of paeoniflorin on cell viability in H₂O₂‐damaged PC12 cells. PC12 cells were treated with 300 μM H₂O₂ and then with various concentrations of paeoniflorin for 24 h. (A). The Chemical Formula of paeoniflorin. (B). Cell viability was assessed by MTT assay. Data are expressed as mean ± SD (*n* = 3). ***p < 0.001 versus Control group; ^#^ *p* < 0.05, ^###^ *p* < 0.001 versus H₂O₂ group.

To investigate the potential protective role of paeoniflorin in SCI, an *in vitro* SCI model was established by treating PC12 cells with 300 μM H₂O₂ for 24 h. After model induction, the cells were further treated with different concentrations of paeoniflorin (25, 50, and 100 μM) for 24 h. Cell viability was assessed using the MTT assay. Figure 1A shows the chemical formula of paeoniflorin. As shown in Figure 1B, compared with the untreated control group, H₂O₂ treatment significantly reduced PC12 cell viability. However, co‐treatment with paeoniflorin led to a concentration‐dependent recovery of cell viability. These results suggest that paeoniflorin confers a protective effect against H₂O₂‐induced cytotoxicity in PC12 cells in a dose‐dependent manner.

### 3.2 Paeoniflorin Inhibits Apoptosis and ROS Accumulation in H₂O₂‐Damaged PC12 Cells

**Figure 2:**
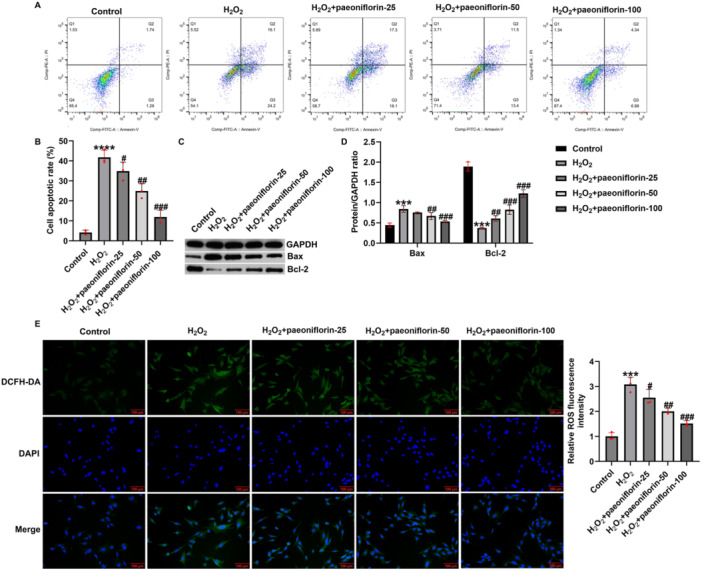
Paeoniflorin inhibits apoptosis and ROS accumulation in H₂O₂‐damaged PC12 cells. (A and B) Flow cytometry analysis of apoptosis rate; (C and D) western blot analysis of Bax and Bcl‐2 protein expression; (E) immunofluorescence detection of intracellular lipid ROS levels (magnification: 200×, bar = 100 μm). Data are presented as mean ± SD (*n* = 3). ***p < 0.001, ****p < 0.0001 versus control group; ^#^ *p* < 0.05, ^##^ *p* < 0.01, ^###^ *p* < 0.001 versus H₂O₂ group.

Oxidative stress and subsequent apoptosis play crucial roles in the secondary injury process in SCI. To further verify the protective effects of paeoniflorin against H₂O₂‐induced damage in PC12 cells, flow cytometry was performed to assess apoptosis. As shown in Figure 2A,B, compared with the control group, H₂O₂ treatment significantly increased the apoptosis rate, whereas paeoniflorin treatment reduced apoptosis in a concentration‐dependent manner. Western blot analysis further confirmed that H₂O₂ markedly upregulated the expression of the pro‐apoptotic protein Bax and downregulated that of the anti‐apoptotic protein Bcl‐2 (Figure 2C,D). Paeoniflorin treatment reversed these effects in a concentration‐dependent manner, with decreased Bax expression and increased Bcl‐2 levels, suggesting that paeoniflorin exerts an anti‐apoptotic effect by modulating the Bax/Bcl‐2 balance.

Immunofluorescence staining revealed that H₂O₂ dramatically elevated intracellular lipid ROS levels, whereas paeoniflorin significantly suppressed ROS accumulation in a dose‐dependent manner (Figure 2E).

These findings demonstrate that paeoniflorin effectively inhibits H₂O₂‐induced apoptosis and ROS accumulation in PC12 cells and exerts neuroprotective effects in a concentration‐dependent manner.

### 3.3 Paeoniflorin Inhibits Ferroptosis in H₂O₂‐Damaged PC12 Cells

**Figure 3:**
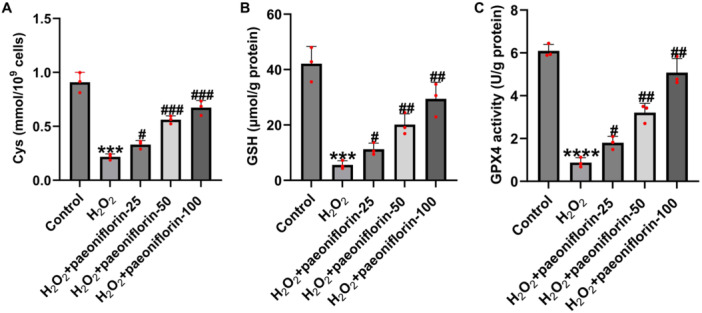
Paeoniflorin inhibits ferroptosis in H₂O₂‐damaged PC12 cells. The expression levels of Cys (A), GSH (B), and GPX4 (C) were measured. H₂O₂ treatment significantly decreased these markers. Paeoniflorin restored Cys, GSH, and GPX4 levels in a concentration‐dependent manner. Data are presented as mean ± SD (*n* = 3). ***p < 0.001, ****p < 0.0001 versus control group; ^#^ *p* < 0.05, ^##^ *p* < 0.01, ^###^ *p* < 0.001 versus H₂O₂ group.

To further investigate the role of paeoniflorin in ferroptosis, we assessed the expression of key ferroptosis‐related metabolites, including Cys, GSH, and GPX4. As shown in Figure 3A–C, H₂O₂ treatment markedly reduced the intracellular levels of Cys, GSH, and GPX4 compared to those in the control group, indicating robust activation of ferroptosis in H₂O₂‐induced PC12 cell injury. Paeoniflorin treatment significantly restored the expression of these metabolites in a dose‐dependent manner. These findings suggest that paeoniflorin exerts neuroprotective effects by alleviating metabolic dysregulation through the modulation of ferroptosis‐associated pathways.

### 3.4 Paeoniflorin Promotes SIRT3 Expression in H₂O₂‐Damaged PC12 Cells

**Figure 4:**
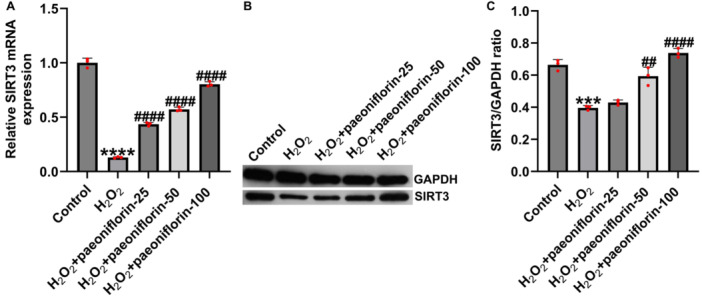
Paeoniflorin promotes SIRT3 expression in H₂O₂‐damaged PC12 cells. (A) RT‐qPCR analysis of SIRT3 mRNA expression; (B and C) western blotting analysis of SIRT3 protein expression. Data are presented as mean ± SD (*n* = 3). ***p < 0.001, ****p < 0.0001 versus control group; ^##^ *p* < 0.01, ^###^ *p* < 0.001 versus H₂O₂ group.

Mitochondrial dysfunction and ROS accumulation play central roles in ferroptosis, while SIRT3, a key mitochondrial deacetylase, is involved in regulating oxidative stress, energy metabolism, and cell survival signaling pathways [29, 30]. Previous studies have confirmed that SIRT3 activation suppresses ferroptosis by reducing ROS levels and stabilizing mitochondrial function [31]. To explore the molecular mechanisms by which paeoniflorin regulates H₂O₂‐induced cellular injury, we assessed the expression of SIRT3, a key regulator of mitochondrial function. SIRT3 is a NAD⁺‐dependent deacetylase involved in maintaining mitochondrial homeostasis, modulating oxidative stress responses, and regulating ferroptosis. As shown in Figure 4A–C, RT‐qPCR and western blotting revealed that H₂O₂ stimulation significantly suppressed SIRT3 mRNA and protein expression compared to the control group. Paeoniflorin treatment effectively restored SIRT3 expression in a concentration‐dependent manner. These results suggest that paeoniflorin can reverse H₂O₂‐induced suppression of SIRT3 expression, providing molecular support for the protective effects against cellular injury.

### 3.5 SIRT3 Inhibitor Suppresses Paeoniflorin‐Induced Upregulation of SIRT3 Expression

**Figure 5:**
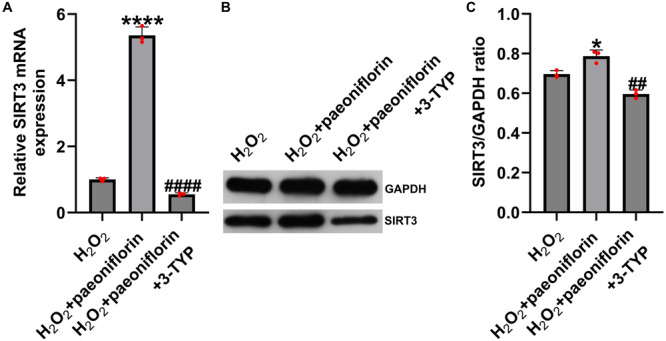
SIRT3 inhibitor 3‐TYP suppresses paeoniflorin‐induced upregulation of SIRT3 expression. (A) RT‐qPCR analysis of SIRT3 mRNA expression; (B and C) western blotting analysis of SIRT3 protein expression. Data are presented as mean ± SD (*n* = 3). *p < 0.05, ****p < 0.0001 versus H₂O₂ group; ^##^ *p* < 0.01, ^####^ *p* < 0.0001 versus H₂O₂ + paeoniflorin group.

To further elucidate whether the protective effect of paeoniflorin on the SCI cell model depends on SIRT3 regulation, PC12 cells were treated with 300 μM H₂O₂ for 24 h to establish an oxidative stress model. Cells were then treated with 100 μM paeoniflorin alone or in combination with the SIRT3 inhibitor 3‐TYP (30 μM) for another 24 h. As shown in Figure 5A–C, RT‐qPCR and western blotting revealed that paeoniflorin significantly increased SIRT3 mRNA and protein expression following H₂O₂ exposure, whereas co‐treatment with 3‐TYP markedly suppressed this upregulation. These findings suggest that 3‐TYP can effectively reverse paeoniflorin‐induced SIRT3 expression in H₂O₂‐treated PC12 cells.

### 3.6 Paeoniflorin Regulates Apoptosis and Ferroptosis in H₂O₂‐Damaged PC12 Cells via Sirt3

**Figure 6:**
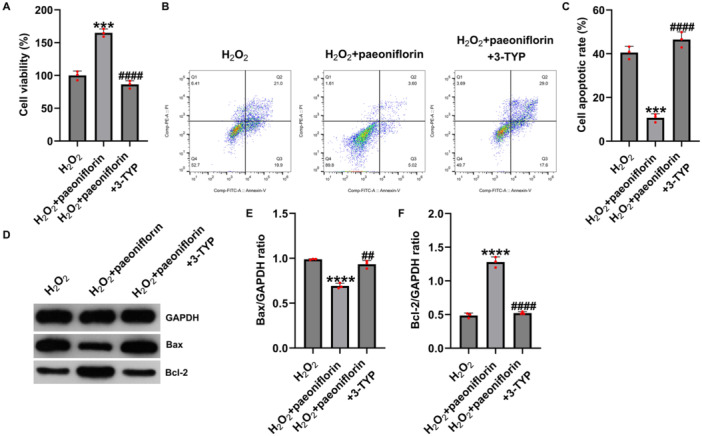
Paeoniflorin regulates apoptosis in H₂O₂‐damaged PC12 cells via SIRT3. (A) Cell viability assessed by MTT assay; (B and C) apoptosis rate analyzed by flow cytometry; (D–F) western blot analysis of Bax and Bcl‐2 protein expression. Data are presented as mean ± SD (*n* = 3). ***p < 0.001, ****p < 0.0001 versus H₂O₂ group; ^##^ *p* < 0.01, ^####^ *p* < 0.0001 versus H₂O₂ + paeoniflorin group.

To further investigate whether SIRT3 mediates the protective effects of paeoniflorin against H₂O₂‐induced cellular injury, we treated PC12 cells with paeoniflorin alone or in combination with the SIRT3‐specific inhibitor, 3‐TYP, and assessed cell viability, apoptosis, oxidative stress, and ferroptosis. MTT assay results showed that H₂O₂ treatment significantly reduced PC12 cell viability, whereas paeoniflorin (100 μM) treatment markedly increased cell viability (Figure 6A). However, co‐treatment with 3‐TYP reduced cell viability (Figure 6A), indicating that SIRT3 inhibition attenuated the protective effects of paeoniflorin.

Flow cytometry further demonstrated that the apoptosis rate increased following H₂O₂ treatment, decreased after paeoniflorin treatment, and increased again after co‐treatment with 3‐TYP (Figure 6B,C). Western blot analysis revealed that paeoniflorin downregulated the pro‐apoptotic protein Bax and upregulated the anti‐apoptotic protein Bcl‐2, whereas 3‐TYP reversed these effects (Figure 6D–F).

**Figure 7:**
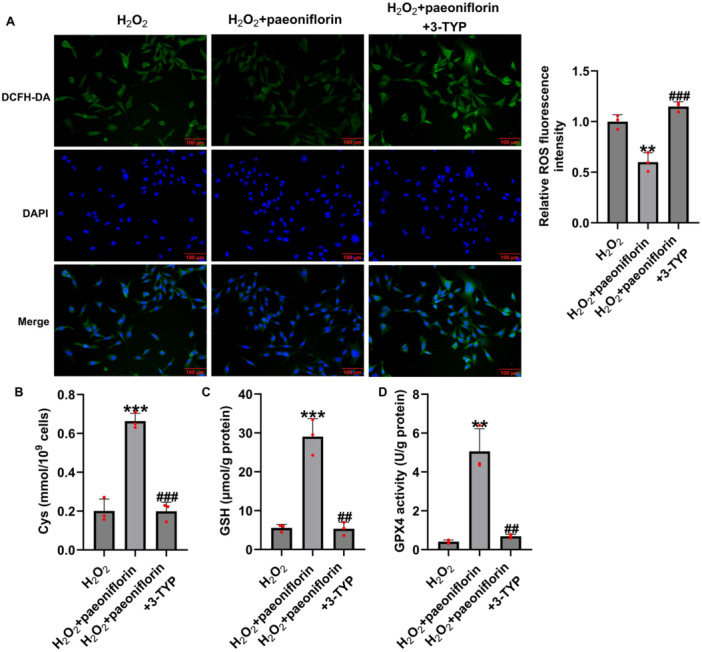
Paeoniflorin regulates ferroptosis in H₂O₂‐damaged PC12 cells via SIRT3. (A) Immunofluorescence detection of intracellular ROS accumulation (Magnification: 200×, bar = 100 μm); (B–D) ELISA assays for Cys, GSH, and GPX4 levels. Data are presented as mean ± SD (*n* = 3). **p < 0.01, ***p < 0.001 versus H₂O₂ group; ^##^ *p* < 0.01, ^###^ *p* < 0.001 versus H₂O₂ + paeoniflorin group.

Immunofluorescence staining showed that paeoniflorin significantly suppressed H₂O₂‐induced ROS accumulation, whereas co‐treatment with 3‐TYP increased ROS levels (Figure 7A). Furthermore, biochemical assays revealed that paeoniflorin markedly increased the levels of Cys, GSH, and GPX4, whereas these levels were partially reduced when 3‐TYP was co‐administered (Figure 7B–D), suggesting that the antiferroptotic effect of paeoniflorin was also compromised.

These findings indicate that paeoniflorin alleviates H₂O₂‐induced cellular damage by activating the SIRT3 pathway and regulating apoptosis, oxidative stress, and ferroptosis.

## 4 Discussion

SCI is a common and severe complication encountered in spinal surgery. Ischemia, hypoxia, and the excessive production of ROS are critical features of secondary injury that lead to mitochondrial dysfunction, lipid peroxidation, and ferroptosis‐mediated cellular damage [10, 32]. Recently, various natural compounds such as tanshinone IIA and astragaloside IV have been shown to alleviate secondary SCI by attenuating oxidative stress and inhibiting cell death pathways [33, 34]. Paeoniflorin, a major bioactive component extracted from *P. lactiflora*, possesses broad neuroprotective effects on the central nervous system and exhibits low toxicity [27]. Previous studies have shown that paeoniflorin effectively delays cellular senescence, exerts anti‐inflammatory and anti‐apoptotic effects, and significantly ameliorates neuronal oxidative stress injury [35]. Wang et al. reported that paeoniflorin exerts neuroprotective effects in SCI models by inhibiting the NF‐κB signaling pathway [36]. However, this is the first study to demonstrate that paeoniflorin protects against SCI by modulating the SIRT3/ROS/ferroptosis axis.

In this study, an oxidative stress injury model was established in PC12 cells by H₂O₂ pretreatment to investigate the effects and underlying mechanisms of paeoniflorin. The results demonstrated that paeoniflorin promoted the proliferation of H₂O₂‐injured PC12 cells, inhibited apoptosis and ROS accumulation in a dose‐dependent manner, and restored the levels of ferroptosis‐related factors, including Cys, GSH, and GPX4. Further investigation revealed that paeoniflorin upregulated the expression of the mitochondrial deacetylase SIRT3, whereas co‐treatment with the SIRT3 inhibitor 3‐TYP significantly attenuated the protective effects of paeoniflorin, suggesting that the SIRT3 signaling pathway plays a crucial role in paeoniflorin‐mediated cytoprotection.

Previous studies have demonstrated that ferroptosis plays a critical role in the secondary injury process following SCI. After SCI, local events such as hemorrhage, iron ion accumulation, plasma membrane rupture, and glutamate excitotoxicity collectively contribute to the activation of ferroptotic pathways [37, 38]. Multiple ferroptosis inhibitors, including ferrostatin‐1 and deferoxamine, have been shown to significantly promote recovery of neurological function after SCI [15, 37, 39]. GPX4, a key regulator of ferroptosis, relies on intracellular GSH levels for its activity, and loss of GPX4 function is considered a pivotal trigger for the onset of ferroptosis [40]. In the present study, paeoniflorin treatment restored the levels of GPX4 and its associated metabolites, suggesting that paeoniflorin may attenuate H₂O₂‐induced cellular injury by suppressing ferroptotic cell death.

Moreover, SIRT3 plays a crucial role in regulating mitochondrial homeostasis, clearing ROS, and maintaining energy metabolism [29, 41]. Studies have demonstrated that SIRT3 enhances ROS scavenging by activating antioxidant enzymes such as manganese superoxide dismutase and catalase, and promotes mitophagy to eliminate damaged mitochondria [42, 43]. In various organ ischemia‐reperfusion injury models, downregulation of SIRT3 has been associated with aggravated cellular damage, whereas its upregulation contributes to improved cell survival and functional recovery [44]. In the present study, paeoniflorin enhanced antioxidant defense and suppressed ferroptosis by upregulating SIRT3 expression, further highlighting the pivotal role of SIRT3 as a potential therapeutic target in SCI.

Although this study demonstrates that paeoniflorin protects against H₂O₂‐induced cellular injury by activating the SIRT3 signaling pathway, certain limitations remain. Because the present study was based on an *in vitro* PC12 cell model, it could not fully replicate the complex *in vivo* microenvironment. Furthermore, whether SIRT3 mediates antioxidant and ferroptosis‐suppressive effects through specific downstream pathways, such as FOXO3a, AMP‐activated protein kinase, and mitophagy, requires further validation in animal models. In addition, the effects of paeoniflorin on other types of neural cells, including microglia and astrocytes, remain unclear. Future studies should aim to evaluate the protective effects and SIRT3 dependency of paeoniflorin in *in vivo* SCI models, explore the potential synergistic effects when combined with therapies, such as hypothermia and iron chelators, and develop nanoparticle‐based delivery systems to enhance its bioavailability and clinical translational potential.

In conclusion, this study confirms that paeoniflorin exerts significant neuroprotective effects by activating the SIRT3 signaling pathway, thereby inhibiting H₂O₂‐induced apoptosis, oxidative stress, and ferroptosis. SIRT3 is a promising molecular target for SCI therapy and may be further developed as a part of future therapeutic strategies for central nervous system injuries.

## Author Contributions

Zongyu Zhang contributed to the study design, data collection, statistical analysis, data interpretation, and manuscript preparation. Zhijing Zhou and Peng Zhang contributed to data collection and statistical analysis. Yongfeng Huo contributed to data collection and manuscript preparation. All authors read and approved the final manuscript.

## Conflicts of Interest

The authors declare no conflicts of interest.

## Data Availability

The datasets used and/or analyzed during the current study are available from the corresponding author on reasonable request.

## References

[1] N. A. Silva , N. Sousa , R. L. Reis , and A. J. Salgado , “From Basics to Clinical: A Comprehensive Review on Spinal Cord Injury,” Progress in Neurobiology 114 (2014): 25–57. 24269804 10.1016/j.pneurobio.2013.11.002

[2] A. Ackery , C. Tator , and A. Krassioukov , “A Global Perspective on Spinal Cord Injury Epidemiology,” Journal of Neurotrauma 21, no. 10 (2004): 1355–1370. 15672627 10.1089/neu.2004.21.1355

[3] M. J. DeVivo , “Epidemiology of Traumatic Spinal Cord Injury: Trends and Future Implications,” Spinal Cord 50, no. 5 (2012): 365–372. 22270188 10.1038/sc.2011.178

[4] J. C. Furlan , B. M. Sakakibara , W. C. Miller , and A. V. Krassioukov , “Global Incidence and Prevalence of Traumatic Spinal Cord Injury,” Canadian Journal of Neurological Sciences Journal Canadien des Sciences Neurologiques 40, no. 4 (2013): 456–464. 10.1017/s0317167100014530 23786727

[5] S. L. James , A. Theadom , R. G. Ellenbogen , et al., “Global, Regional, and National Burden of Traumatic Brain Injury and Spinal Cord Injury, 1990–2016: A Systematic Analysis for the Global Burden of Disease Study 2016,” Lancet Neurology 18, no. 1 (2019): 56–87. 30497965 10.1016/S1474-4422(18)30415-0 PMC6291456

[6] J. W. Rowland , G. W. J. Hawryluk , B. Kwon , and M. G. Fehlings , “Current Status of Acute Spinal Cord Injury Pathophysiology and Emerging Therapies: Promise on the Horizon,” Neurosurgical Focus 25, no. 5 (2008): E2. 10.3171/FOC.2008.25.11.E2 18980476

[7] W. Tetzlaff , E. B. Okon , S. Karimi‐Abdolrezaee , et al., “A Systematic Review of Cellular Transplantation Therapies for Spinal Cord Injury,” Journal of Neurotrauma 28, no. 8 (2011): 1611–1682. 20146557 10.1089/neu.2009.1177 PMC3143488

[8] C. Oyinbo , “Secondary Injury Mechanisms in Traumatic Spinal Cord Injury: A Nugget of This Multiply Cascade,” Acta Neurobiologiae Experimentalis 71, no. 2 (2011): 281–299. 21731081 10.55782/ane-2011-1848

[9] C. S. Ahuja and M. Fehlings , “Concise Review: Bridging the Gap: Novel Neuroregenerative and Neuroprotective Strategies in Spinal Cord Injury,” Stem Cells Translational Medicine 5, no. 7 (2016): 914–924. 27130222 10.5966/sctm.2015-0381 PMC4922857

[10] E. D. Hall and J. E. Springer , “Neuroprotection and Acute Spinal Cord Injury: A Reappraisal,” NeuroRx 1, no. 1 (2004): 80–100. 15717009 10.1602/neurorx.1.1.80 PMC534914

[11] B. R. Stockwell , J. P. Friedmann Angeli , H. Bayir , et al., “Ferroptosis: A Regulated Cell Death Nexus Linking Metabolism, Redox Biology, and Disease,” Cell 171, no. 2 (2017): 273–285, 10.1016/j.cell.2017.09.021. 28985560 PMC5685180

[12] Y. Xie , W. Hou , X. Song , et al., “Ferroptosis: Process and Function,” Cell Death and Differentiation 23, no. 3 (2016): 369–379. 26794443 10.1038/cdd.2015.158 PMC5072448

[13] C. Wu , W. Zhao , J. Yu , S. Li , L. Lin , and X. Chen , “Induction of Ferroptosis and Mitochondrial Dysfunction by Oxidative Stress in PC12 Cells,” Scientific Reports 8, no. 1 (2018): 574. 29330409 10.1038/s41598-017-18935-1 PMC5766540

[14] Y. Yu , Y. Yan , F. Niu , et al., “Ferroptosis: A Cell Death Connecting Oxidative Stress, Inflammation and Cardiovascular Diseases,” Cell Death Discovery 7, no. 1 (2021): 193. 34312370 10.1038/s41420-021-00579-w PMC8313570

[15] H. Ge , X. Xue , J. Xian , et al., “Ferrostatin‐1 Alleviates White Matter Injury via Decreasing Ferroptosis Following Spinal Cord Injury,” Molecular Neurobiology 59, no. 1 (2022): 1–16. 10.1007/s12035-021-02571-y 34635980

[16] J. Song , Y. Liu , Y. Guo , et al., “Therapeutic Effects of Tetrandrine in Inflammatory Diseases: A Comprehensive Review,” Inflammopharmacology 32, no. 3 (2024): 1743–1757. 38568399 10.1007/s10787-024-01452-9

[17] L. Zhang and W. Wei , “Anti‐Inflammatory and Immunoregulatory Effects of Paeoniflorin and Total Glucosides of Paeony,” Pharmacology & Therapeutics 207 (2020): 107452. 31836457 10.1016/j.pharmthera.2019.107452

[18] X.‐X. Zhang , J.‐Q. Zuo , Y.‐T. Wang , H.‐Y. Duan , J.‐H. Yuan , and Y.‐H. Hu , “Paeoniflorin in Paeoniaceae: Distribution, Influencing Factors, and Biosynthesis,” Frontiers in Plant Science 13 (2022): 980854. 36119574 10.3389/fpls.2022.980854 PMC9478390

[19] H. W. Meng , A. Y. Lee , H. Y. Kim , E. J. Cho , and J. H. Kim , “Neuroprotective Effects of Paeoniflorin Against Neuronal Oxidative Stress and Neuroinflammation Induced by Lipopolysaccharide in Mice,” Journal of Applied Biological Chemistry 65, no. 1 (2022): 23–31.

[20] Y. Zhang , L. L. Wang , Y. Wu , et al., “Paeoniflorin Attenuates Hippocampal Damage in a Rat Model of Vascular Dementia,” Experimental and Therapeutic Medicine 12, no. 6 (2016): 3729–3734. 28101164 10.3892/etm.2016.3849 PMC5228285

[21] Z. He , P. Huan , L. Wang , and J. He , “Paeoniflorin Ameliorates Cognitive Impairment in Parkinson's Disease via JNK/p53 Signaling,” Metabolic Brain Disease 37, no. 4 (2022): 1057–1070. 35230626 10.1007/s11011-022-00937-2 PMC9042992

[22] X. Gu , Z. Cai , M. Cai , et al., “Protective Effect of Paeoniflorin on Inflammation and Apoptosis in the Cerebral Cortex of a Transgenic Mouse Model of Alzheimer's Disease,” Molecular Medicine Reports 13, no. 3 (2016): 2247–2252. 26796245 10.3892/mmr.2016.4805

[23] R.‐B. Guo , G.‐F. Wang , A.‐P. Zhao , J. Gu , X.‐L. Sun , and G. Hu , “Paeoniflorin Protects Against Ischemia‐Induced Brain Damages in Rats via Inhibiting MAPKs/NF‐κB‐Mediated Inflammatory Responses,” PLoS One 7, no. 11 (2012): e49701. 23166749 10.1371/journal.pone.0049701 PMC3498223

[24] H. Liu , J. Wang , J. Wang , P. Wang , and Y. Xue , “Paeoniflorin Attenuates Aβ1‐42‐Induced Inflammation and Chemotaxis of Microglia In Vitro and Inhibits NF‐κB‐and VEGF/Flt‐1 Signaling Pathways,” Brain Research 1618 (2015): 149–158. 26049130 10.1016/j.brainres.2015.05.035

[25] F. Chen , H.‐T. Lu , and Y. Jiang , “Purification of Paeoniflorin From Paeonia lactiflora Pall. by High‐Speed Counter‐Current Chromatography,” Journal of Chromatography A 1040, no. 2 (2004): 205–208. 15230527 10.1016/j.chroma.2004.04.023

[26] H. Liu , C. Yu , T. Xu , X. Zhang , and M. Dong , “Synergistic Protective Effect of Paeoniflorin and β‐Ecdysterone Against Rotenone‐Induced Neurotoxicity in PC12 Cells,” Apoptosis 21 (2016): 1354–1365. 27688248 10.1007/s10495-016-1293-7

[27] P. Liu , J. Cheng , S. Ma , and J. Zhou , “Paeoniflorin Attenuates Chronic Constriction Injury‐Induced Neuropathic Pain by Suppressing Spinal NLRP3 Inflammasome Activation,” Inflammopharmacology 28, no. 6 (2020): 1495–1508, 10.1007/s10787-020-00737-z. 32596762

[28] K. J. Livak and T. D. Schmittgen , “Analysis of Relative Gene Expression Data Using Real‐Time Quantitative PCR and the 2^−ΔΔCT^ Method,” Methods 25, no. 4 (2001): 402–408, 10.1006/meth.2001.1262. 11846609

[29] D. B. Lombard , F. W. Alt , H.‐L. Cheng , et al., “Mammalian Sir2 Homolog SIRT3 Regulates Global Mitochondrial Lysine Acetylation,” Molecular and Cellular Biology 27, no. 24 (2007): 8807–8814. 17923681 10.1128/MCB.01636-07 PMC2169418

[30] A. H. H. Tseng , S.‐S. Shieh , and D. L. Wang , “SIRT3 Deacetylates FOXO3 to Protect Mitochondria Against Oxidative Damage,” Free Radical Biology and Medicine 63 (2013): 222–234. 23665396 10.1016/j.freeradbiomed.2013.05.002

[31] J. Zheng , L. Shi , F. Liang , et al., “SIRT3 Ameliorates Oxidative Stress and Mitochondrial Dysfunction After Intracerebral Hemorrhage in Diabetic Rats,” Frontiers in Neuroscience 12 (2018): 414. 29970985 10.3389/fnins.2018.00414 PMC6018086

[32] F. Bao and D. Liu , “Peroxynitrite Generated in the Rat Spinal Cord Induces Neuron Death and Neurological Deficits,” Neuroscience 115, no. 3 (2002): 839–849. 12435422 10.1016/s0306-4522(02)00506-7

[33] X.‐M. Zhang , J. Ma , Y. Sun , et al., “Tanshinone IIA Promotes the Differentiation of Bone Marrow Mesenchymal Stem Cells Into Neuronal‐Like Cells in a Spinal Cord Injury Model,” Journal of Translational Medicine 16 (2018): 193. 30001730 10.1186/s12967-018-1571-y PMC6044071

[34] Y. Zhou , L. Li , C. Mao , and D. Zhou , “Astragaloside IV Ameliorates Spinal Cord Injury Through Controlling Ferroptosis in H2O2‐Damaged PC12 Cells In Vitro,” Annals of Translational Medicine 10, no. 21 (2022): 1176, 10.21037/atm-22-5196. 36467371 PMC9708485

[35] J. Zhou , J. Wang , W. Li , C. Wang , L. Wu , and J. Zhang , “Paeoniflorin Attenuates the Neuroinflammatory Response in a Rat Model of Chronic Constriction Injury,” Molecular Medicine Reports 15, no. 5 (2017): 3179–3185, 10.3892/mmr.2017.6371. 28339077

[36] B. Wang , W. Dai , L. Shi , et al., “Neuroprotection by Paeoniflorin Against Nuclear Factor Kappa B‐Induced Neuroinflammation on Spinal Cord Injury,” BioMed Research International 2018 (2018): 9865403, 10.1155/2018/9865403. 30627586 PMC6304651

[37] X. Yao , Y. Zhang , J. Hao , et al., “Deferoxamine Promotes Recovery of Traumatic Spinal Cord Injury by Inhibiting Ferroptosis,” Neural Regeneration Research 14, no. 3 (2019): 532–541. 30539824 10.4103/1673-5374.245480 PMC6334606

[38] Q. Li , X. Han , X. Lan , et al., “Inhibition of Neuronal Ferroptosis Protects Hemorrhagic Brain,” JCI Insight 2, no. 7 (2017): e90777. 28405617 10.1172/jci.insight.90777 PMC5374066

[39] H. Jia , X. Liu , Y. Cao , et al., “Deferoxamine Ameliorates Neurological Dysfunction by Inhibiting Ferroptosis and Neuroinflammation After Traumatic Brain Injury,” Brain Research 1812 (2023): 148383. 37149247 10.1016/j.brainres.2023.148383

[40] B. R. Stockwell , J. P. Friedmann Angeli , H. Bayir , et al., “Ferroptosis: A Regulated Cell Death Nexus Linking Metabolism, Redox Biology, and Disease,” Cell 171, no. 2 (2017): 273–285. 28985560 10.1016/j.cell.2017.09.021 PMC5685180

[41] Y. Chen , J. Zhang , Y. Lin , et al., “Tumour Suppressor SIRT3 Deacetylates and Activates Manganese Superoxide Dismutase to Scavenge Ros,” EMBO Reports 12, no. 6 (2011): 534–541. 21566644 10.1038/embor.2011.65 PMC3128277

[42] R. Tao , A. Vassilopoulos , L. Parisiadou , Y. Yan , and D. Gius , “Regulation of MnSOD Enzymatic Activity by Sirt3 Connects the Mitochondrial Acetylome Signaling Networks to Aging and Carcinogenesis,” Antioxidants & Redox Signaling 20, no. 10 (2014): 1646–1654. 23886445 10.1089/ars.2013.5482 PMC3942696

[43] G. P. Maiti , S. Sinha , H. Mahmud , et al., “SIRT3 Overexpression and Epigenetic Silencing of Catalase Regulate ROS Accumulation in CLL Cells Activating AXL Signaling Axis,” Blood Cancer Journal 11, no. 5 (2021): 93. 34001853 10.1038/s41408-021-00484-6 PMC8129117

[44] C. Gu , F. Kong , J. Zeng , X. Geng , Y. Sun , and X. Chen , “Remote Ischemic Preconditioning Protects Against Spinal Cord Ischemia–Reperfusion Injury in Mice by Activating NMDAR/AMPK/PGC‐1α/SIRT3 Signaling,” Cell & Bioscience 13, no. 1 (2023): 57. 36927808 10.1186/s13578-023-00999-4 PMC10018930

